# Effect of a Virtual Home-Based Behavioral Intervention on Family Health and Resilience During the COVID-19 Pandemic: A Randomized Clinical Trial

**DOI:** 10.1001/jamanetworkopen.2022.47691

**Published:** 2022-12-20

**Authors:** Filoteia Popescu, Evan C. Sommer, Margaret R. Mahoney, Laura E. Adams, Shari L. Barkin

**Affiliations:** 1 The University of Tennessee Health Science Center College of Medicine, Memphis; 2 Department of Academic General Pediatrics, Vanderbilt University Medical Center, Nashville, Tennessee; 3 Vanderbilt University School of Medicine, Nashville, Tennessee; 4 Department of Pediatrics, Children’s Hospital of Richmond at Virginia Commonwealth University, Richmond

## Abstract

**Importance:**

Virtual home-based interventions may bolster protective factors, such as family health and resilience, during stressors such as the COVID-19 pandemic; however, their effectiveness is unknown.

**Objective:**

To examine the effectiveness of a virtual health coaching intervention on family health and resilience during the pandemic.

**Design, Setting, and Participants:**

In this parallel-group, single-site randomized clinical trial, 123 parents and their 2- to 8-year-old children were enrolled at a pediatric clinic or community partner site in Tennessee from March 10 to August 11, 2021. Follow-up surveys were completed between June 29 and November 11, 2021.

**Interventions:**

All participants received 11 weekly cooking videos and associated home-delivered groceries. The intervention group also received 12 weekly, 30-minute virtual health coach sessions.

**Main Outcomes and Measures:**

The primary outcome was the validated 6-item (range, 6-30) Family Healthy Lifestyle Subscale (FHLS) scores. The secondary outcome was the validated 6-item (range, 0-6) Family Resilience and Connection Index (FRCI) scores. Outcomes were determined a priori and evaluated at baseline and 12-week follow-up. A priori independent *t* tests and multivariable tobit regression models assessed intervention effects, and post hoc, secondary interaction models assessed whether effects differed over baseline outcomes.

**Results:**

Among the 123 enrolled families, 110 (89%) were included in the primary analyses (parent mean [SD] age, 35.1 [8.2] years; 104 [95%] female; 55 [50%] non-Hispanic Black; child mean [SD] age, 5.2 [1.7] years; 62 [56%] male). Intervention-control group mean differences were nonsignificant for follow-up FHLS scores (0.7; 95% CI, −0.6 to 2.0; *P* = .17) and FRCI scores (0.1; 95% CI, −0.5 to 0.6; *P* = .74). Tobit regression model intervention effects were nonsignificant for FHLS scores (0.9; 95% CI, −0.3 to 2.2; *P* = .15) and FRCI scores (0.4; 95% CI, −0.2 to 1.1; *P* = .17). Post hoc, secondary models found no significant interaction for FHLS scores (1.3 increase per 5-point decrease; 95% CI, −0.2 to 2.7; *P* = .09), with significant intervention associations for baseline scores of 6 to 23. The interaction was significant for FRCI scores (0.4 increase per 1-point decrease; 95% CI, 0.01 to 0.8; *P* = .047), with significant intervention associations for baseline scores of 0 to 3.

**Conclusions and Relevance:**

In this randomized clinical trial of families with young children, weekly virtual health coaching did not detectably improve family health and resilience. Post hoc, secondary results provided preliminary evidence of potential effectiveness among families with low baseline scores.

**Trial Registration:**

ClinicalTrials.gov Identifier: NCT05328193

## Key Points

**Question:**

How did a virtual health coaching intervention affect family health and resilience during the COVID-19 pandemic?

**Findings:**

In this randomized clinical trial, all 123 parent-child dyads received weekly cooking videos and associated groceries. Randomization to receive an additional weekly virtual health coaching intervention did not cause significant improvements in family health or resilience compared with the control group; however, post hoc, secondary interaction models estimated a significant association among those with lower baseline scores.

**Meaning:**

Although there were no detectable outcome differences between groups, post hoc analyses suggest the intervention may have been effective among families with low baseline scores.

## Teaser

This randomized clinical trial evaluates the effectiveness of a virtual health coaching intervention in diverse, underserved families with young children during the COVID-19 pandemic.

## Introduction

Families with young children are acutely vulnerable to the social, economic, and health-related challenges exacerbated by the COVID-19 pandemic.^1,2,3,4^ Although Tennessee’s stay-at-home order ended in April 2020, families continued to experience significant disruptions to their daily routines as many schools and childcare centers were shut down or operating remotely throughout 2021.^5,6^ Children had reduced in-person contact with their peers, contributing to feelings of boredom, frustration, and loneliness.^7,8,9^ Moreover, children’s health behaviors were adversely affected, resulting in increased sedentary behavior, disrupted sleep patterns, and poor eating habits.^10,11,12,13^ Many parents also faced heightened responsibility with their children at home, increasing caregiver burnout.^14,15^

Studies suggest that strong family health and resilience can be protective, buffering against stresses.^16,17,18,19^ Healthy family behaviors, such as shared meals, are associated with better nutrition, improved parent-child communication, and lower rates of depression and anxiety.^20,21,22,23^ Likewise, strong family resilience, defined as a family’s ability to effectively cope, adapt, and grow together when encountering stressors,^24^ promotes children’s well-being during adversity and may have long-lasting effects in adulthood (eg, a reduced risk of smoking, intergenerational child maltreatment, and major chronic diseases).^25,26,27,28,29,30,31,32^

Although many interventions focus on children or parents, few studies measure family health. This randomized clinical trial (RCT) aimed to address this gap by evaluating the effectiveness of a 12-week Virtual Healthier Families (VHF) program in a sample of diverse, underserved families with young children during COVID-19.^33,34,35,36^ All families who participated in the study received a Virtual Teaching Kitchen Outreach (VTKO) program (weekly cooking videos and associated home-delivered groceries),^37^ and families randomized to the intervention group also received the VHF program (weekly virtual health coaching sessions). Families who received the VHF program were hypothesized to have improved family health and resilience after the intervention compared with families who only received the VTKO program.

## Methods

### Study Design and Population

This parallel-group, single-site RCT was approved by the Vanderbilt Institutional Review Board. The protocol is available in Supplement 1. This study followed the Consolidated Standards of Reporting Trials (CONSORT) guidelines. In error, this study was not registered before participant enrollment; however, this study was retrospectively registered at ClinicalTrials.gov.

Parent-child dyads were recruited on a rolling basis from the Vanderbilt Pediatric Primary Care Clinic and 3 community partner sites in Nashville, Tennessee. Recruitment efforts included flyers, telephone calls, text messages, and emails. Families were included if the parent was 18 years or older, provided written informed consent, spoke English, and had a 2- to 8-year-old child. There were no prespecified health or behavioral factors that affected eligibility. For families with multiple eligible children, the participating parent specified which child was the index child at baseline. Sibling information was not recorded. Families were excluded if they could not participate for 12 weeks, lived outside the grocery delivery service area, or lacked access to a device that could receive the VTKO or VHF programs. Families were enrolled from March 10 to August 11, 2021. Follow-up surveys were conducted from June 29 to November 11, 2021. All data, including participant characteristics, were self-reported. Data on race, ethnicity, and other sociodemographic factors were collected to further describe the participants in the study and provide information about the potential generalizability of the results. Participants were provided gift cards up to $30 total for completing all surveys.

### Randomization and Blinding

After baseline data collection, participants were randomized to the control (VTKO only) or intervention group (VTKO plus VHF programming) using a computer-generated schedule with randomly permuted block sizes of 2 or 4. Assignment was balanced across recruitment sites. An electronic interface concealed upcoming assignment before enrollment, and assignment could not be changed. A statistician (E.C.S.) generated and had sole access to the randomization schedule. Only blinded research staff (F.P. and M.R.M.) collected data.

### Intervention Description

The 12-week VTKO and VHF programs are adapted from previously tested in-person programs.^33,34,35,37^ In the current study, all enrolled families received the VTKO program, which included a weekly text message with the link to a short, easy-to-follow cooking video for a healthy, low-cost recipe, such as zucchini pizza boats and banana oatmeal cookies. All ingredients could be purchased using Supplemental Nutrition Assistance Program (SNAP) and the Special Supplemental Nutrition Program for Women, Infants and Children (WIC). Recipes required approximately 20 minutes to prepare. Weekly home-delivered groceries were provided for each recipe.

In addition to the VTKO program, families randomized to the intervention group received the VHF program. Each parent-child dyad met with a trained health coach for a weekly, 30-minute session using Zoom (Zoom Video Communications Inc). Participants were assigned to a health coach based on participants’ availability. The purpose of the VHF program was to help families build sustainable, healthy behavior skills regarding nutrition, physical activity, and general family wellness. Session topics included Choose Healthy Foods and Engaged Parenting (eTable 1 in Supplement 2). Health coaches shared their screen to present a standard curriculum developed with health literacy and childhood obesity experts.^35^ Each session included (1) goal check-in with problem-solving, (2) why the topic is important for family health, (3) interactive didactic, (4) a hands-on, skills-building activity, and (5) goal setting. Because all participants were randomized and received the VTKO program, any observed effects can be causally attributed to the VHF program.

### Outcomes

Primary and secondary outcomes were specified a priori. Data were collected at baseline (before randomization) and 12-week follow-up (after intervention) using REDCap (Research Electronic Data Capture).^38,39^ Surveys were self-administered online or collected over the telephone with a trained data collector (F.P. and M.R.M.).

The primary outcome, family health, was measured using the validated 6-item Family Healthy Lifestyle Subscale (FHLS) (eTable 2 in Supplement 2).^40^ Although the term *family health* lacks an established definition, the FHLS was chosen because it most closely aligned with the family health behaviors the VHF intervention aimed to change. Prior studies also demonstrated high internal consistency (Cronbach α = 0.82-0.90).^40,41^ Responses range from 1 (strongly disagree) to 5 (strongly agree). Total scores range from 6 to 30, with higher scores indicating better family health.

The secondary outcome, family resilience, was measured using the validated 6-item Family Resilience and Connection Index (FRCI) (eTable 3 in Supplement 2).^42^ A prior study demonstrated high internal consistency of the FRCI (Cronbach α = 0.85).^43^ For the first 4 items, responses range from 0 (none of the time) to 1 (all of the time). For the last 2 items, responses range from 0 (not very well or not at all) to 1 (very well). Total scores range from 0 to 6, with higher scores indicating stronger family resilience.

### Statistical Analysis

Because the newly validated FHLS had not been widely studied at the time of study conception, little information was available for an a priori power analysis. Therefore, a post hoc power analysis based on the current study’s data was conducted to assess whether the obtained sample size could detect a meaningful effect using the following assumptions: a 2-tailed significance level α < .05, the baseline FHLS covariate explaining approximately 10% of follow-up FHLS variance, and an analytic sample size of 110. The power analysis estimated that the smallest standardized effect size detectable with 80% power was 0.51, or approximately half an SD. On the basis of the observed overall follow-up FHLS SD (2.7), this translates to detecting an approximate intervention effect as small as 1.4 points.

Participant characteristics are presented as means (SDs) for continuous variables and numbers (percentages) for categorical variables. An intention-to-treat approach was used to compare outcomes in a priori analyses. Outcome means were initially compared using independent *t* tests. Due to a substantial degree of left skew and possible ceiling effect on the outcome measures at follow-up, the intervention effect was assessed via a multivariable tobit regression model with upper limits of 30 for FHLS and 6 for FRCI. The primary regression model adjusted for baseline score, random assignment, marital status, parental educational level, and parent race and ethnicity. A post hoc, secondary tobit regression model used the same covariates as the primary model and included an interaction between baseline score and random assignment to determine whether the intervention may have been more effective for families with lower baseline scores. To facilitate interpretation of the interaction results, plots were generated showing model-based estimates of the intervention’s effect on follow-up score over the full range of baseline scores. Baseline scores for which the intervention effect’s 95% CI did not include zero indicate statistical significance. Additional plots show the estimated follow-up scores by study group over baseline scores. However, these results should be interpreted with caution because these analyses were not prespecified.

All reported *P* values were 2-sided, with statistical significance defined as *P* < .05. Statistical and power analyses were conducted using Stata software, version 17.0 (StataCorp LLC) and Optimal Design, version 3.01, respectively.^44^

## Results

**Figure 1.**
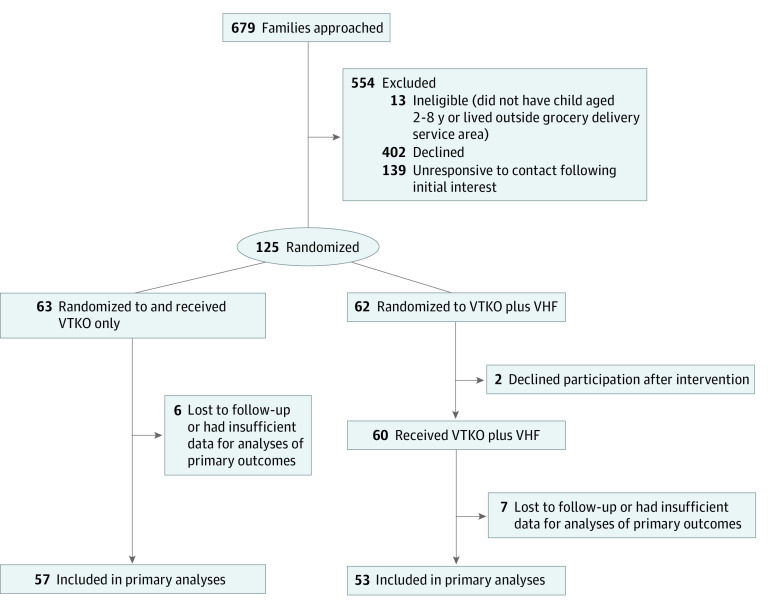
Study Flow Diagram VHF indicates Virtual Healthier Families; VTKO, Virtual Teaching Kitchen Outreach.

Figure 1 presents the CONSORT diagram. Of 679 families approached, 402 (59%) declined participation mostly due to time constraints (eg, work schedule). Thirteen families (2%) were ineligible due to location or age exclusion criteria. No families lacked access to a device that could receive the VTKO or VHF programs. Although 125 families (18%) were randomized (63 to the control group and 62 to the intervention group), 2 families declined participation immediately following randomization and did not receive the VTKO or VHF program. These 2 withdrawn families were excluded from all results. No significant demographic differences were found between enrolled participants who were included or excluded from analysis (eTable 4 in Supplement 2). There was also no differential attrition.

### Baseline Characteristics

Table 1 provides the participant characteristics. Child mean (SD) age was 5.2 (1.7) years, 62 (56%) were male, and 48 (44%) were female. Parent mean (SD) age was 35.1 (8.2) years, 104 (95%) were female, and 6 (5%) were male. Twelve parents (11%) reported being Hispanic of any race, 55 (50%) were non-Hispanic Black, 31 (28%) were non-Hispanic White, and 12 (11%) were of other race or ethnicity, multiracial, or multiethnic. Most families represented disadvantaged groups, with 62 (56%) reporting SNAP and/or WIC use and 29 (26%) reporting food insecurity or hunger. There were no significant differences between study groups.

**Table 1.** Participant Characteristics^a^

Characteristic	Control group (VTKO only) (n = 57)	Intervention group (VTKO and VHF) (n = 53)	Total (N = 110)
**Child**			
Age, mean (SD), y	4.9 (1.7)	5.5 (1.6)	5.2 (1.7)
Sex			
Male	35 (61)	27 (51)	62 (56)
Female	22 (39)	26 (49)	48 (44)
**Parent**			
Age, mean (SD), y	34.0 (7.0)	36.1 (9.2)	35.1 (8.2)
Sex			
Male	2 (4)	4 (8)	6 (5)
Female	55 (96)	49 (92)	104 (95)
Race and ethnicity^b^			
Hispanic, any race	5 (9)	7 (13)	12 (11)
Non-Hispanic Black	26 (46)	29 (55)	55 (50)
Non-Hispanic White	20 (35)	11 (21)	31 (28)
Other or multiracial or multiethnic^c^	6 (11)	6 (11)	12 (11)
Educational level			
High school or less	13 (23)	16 (30)	29 (26)
More than high school	44 (77)	37 (70)	81 (74)
Marital status			
Single, divorced, separated, or widowed	29 (51)	31 (58)	60 (55)
Married or living as couple	28 (49)	22 (42)	50 (45)
**Family**			
No. of children in household			
1	17 (30)	14 (26)	31 (28)
>1	40 (70)	19 (74)	79 (72)
No. of adults in household			
1	23 (40)	18 (34)	41 (37)
>1	34 (60)	35 (66)	69 (63)
Household food security			
Food secure	41 (72)	40 (75)	81 (74)
Food insecure with or without hunger	16 (28)	13 (25)	29 (26)
Use of SNAP and/or WIC			
No	27 (47)	21 (40)	48 (44)
Yes	30 (53)	32 (60)	62 (56)
Annual household income, $			
<10 000	8 (14)	13 (25)	21 (19)
10 000-19 999	5 (9)	4 (8)	9 (8)
20 000-34 999	12 (21)	9 (17)	21 (19)
35 000-49 999	9 (16)	9 (17)	18 (16)
50 000-74 999	6 (11)	5 (9)	11 (10)
75 000-99 999	1 (2)	2 (4)	3 (3)
≥100 000	6 (11)	2 (4)	8 (7)
Do not know or prefer not to answer	10 (18)	9 (17)	19 (17)

Abbreviations: SNAP, Supplemental Nutrition Assistance Program; VHF, Virtual Healthier Families; VTKO, Virtual Teaching Kitchen Outreach; WIC, Special Supplemental Nutrition Program for Women, Infants, and Children.

^a^ Data are presented as number (percentage) of study participants unless otherwise indicated. No significant differences were found between the control and intervention groups at baseline for any of the reported participant demographic characteristics. Differences between study groups were calculated using a 2-sample independent *t* test for continuous variables and a χ^2^ test for independence for categorical variables.

^b^ Race and ethnicity classifications were aligned with research-based recommendations from the US Census. All participant characteristics, including parent race and ethnicity, were collected through parent self-report.

^c^ After data cleaning, the “other” category, by itself, was selected by 1 participant who chose not to further specify their race or ethnicity. Additional racial or ethnic responses that were categorized as other included Asian, Middle Eastern, Native American, Native Hawaiian, and Pacific Islander (n = 3). The multiracial or multiethnic category included any participants who indicated more than 1 race or ethnicity (n = 8). Because of the small sample sizes, these 2 categories were combined into the "other or multiracial or multiethnic" category for analysis.

### Process Measures

Of the 110 families included in primary analyses, 106 (96%) received all 11 VTKO cooking videos and associated groceries. Exceptions were due to food allergies or a change in home address. A total of 108 families (98%) responded to process measure survey items, reporting a mean (SD) number of 9 (3) recipes prepared. Of the 53 intervention group families included in primary analyses, the mean (SD) number of VHF sessions attended was 6 (5).

### Primary Outcome

A total of 110 families (89%) had complete FHLS baseline and follow-up data for analysis. Baseline FHLS scores (range, 6-30) were left-skewed, with reported means (SDs) of 25.4 (4.4) for the control group and 25.6 (4.1) for the intervention groups (Table 2). Although 12-week follow-up mean (SD) FHLS scores were descriptively higher in both groups (27.0 [3.0] in the control group and 27.7 [2.2] in the intervention group), the difference between groups was not statistically significant (0.7; 95% CI, −0.6 to 2.0; *P* = .17). Internal consistency of the FHLS was relatively high (Cronbach α = 0.82 at baseline and 0.77 at 12-week follow-up).

**Table 2.** Comparison of Mean Outcome Scores Between Study Groups

Outcome	Mean (SD) score		Between-group difference, mean (95% CI)
	Control group (VTKO only)	Intervention group (VTKO plus VHF)	
**FHLS^a^**			
Baseline	25.4 (4.4)	25.6 (4.1)	NA
12-wk Follow-up	27.0 (3.0)	27.7 (2.2)	0.7 (−0.6 to 2.0)
**FRCI^b^**			
Baseline	4.7 (1.3)	4.3 (1.8)	NA
12-wk Follow-up	4.4 (1.5)	4.5 (1.5)	0.1 (−0.5 to 0.6)

Abbreviations: FHLS, Family Healthy Lifestyle Subscale; FRCI, Family Resilience and Connection Index; NA, not applicable; VHF, Virtual Healthier Families; VTKO, Virtual Teaching Kitchen Outreach.

^a^ The FHLS total score ranges from 6 to 30. A total of 110 participants (57 in the control group and 53 in the intervention group) were included in baseline and follow-up FHLS total score primary analyses.

^b^ The FRCI total score ranges from 0 to 6. Three additional participants had sufficient data for baseline and follow-up FRCI total score secondary analyses. Therefore, a sample size of 113 (59 in the control group and 54 in the intervention group) was reported for all FRCI results.

**Figure 2.**
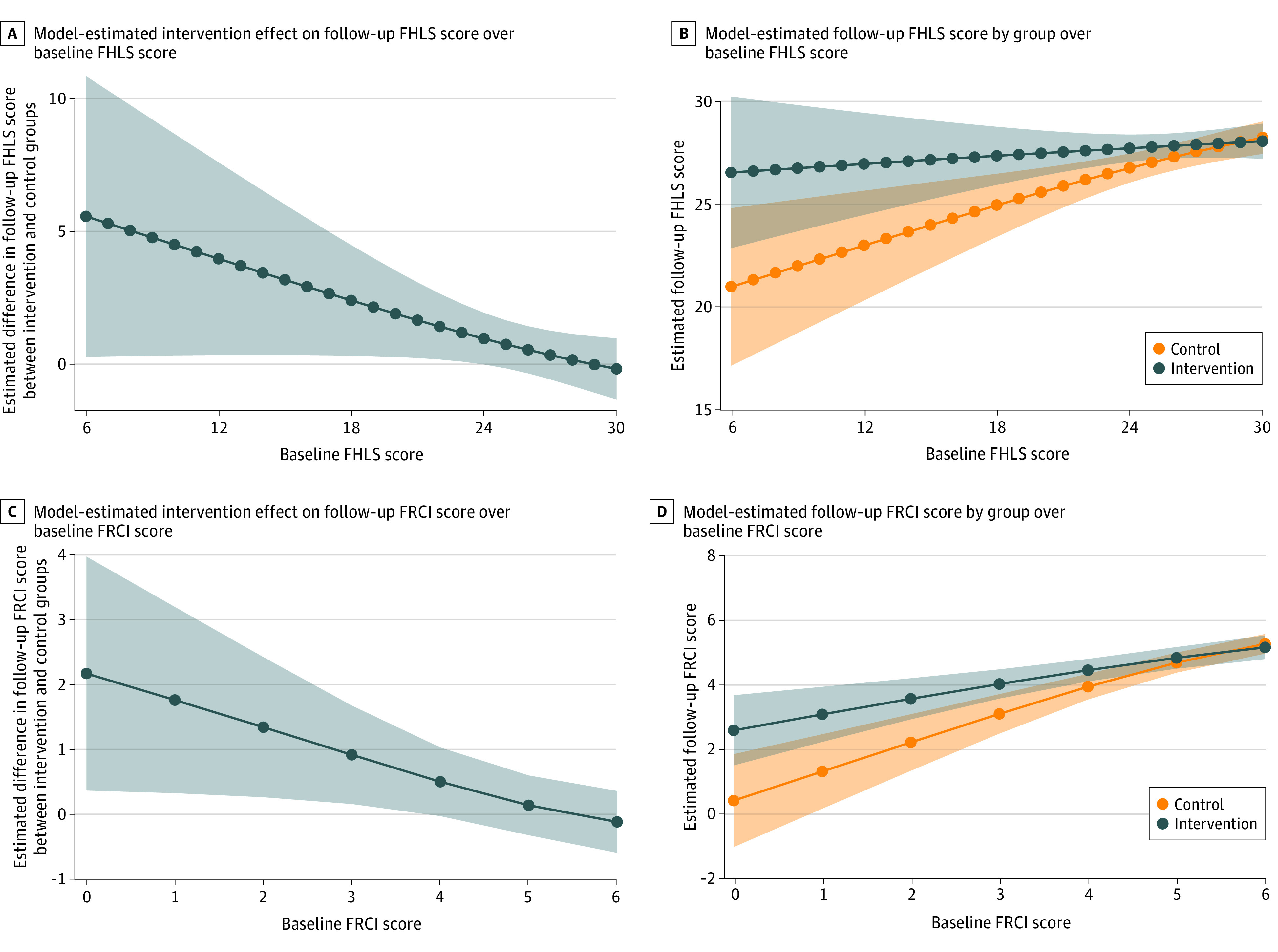
Interaction Model–Based Estimates of Follow-up Outcome Score by Group Over Baseline Score Model-based estimates are from a tobit regression model adjusting for baseline outcome score (Family Healthy Lifestyle Subscale [FHLS] or Family Resilience and Connection Index [FRCI]), random assignment, and their interaction, as well as marital status, parental educational level, and parent race and ethnicity. Plotted lines represent estimates of follow-up outcome (FHLS or FRCI) scores for the intervention and control groups over different baseline outcome scores. Shaded areas indicate 95% CIs. To reflect the upper bound imposed by the tobit regression model, estimates were constrained from exceeding the respective upper bounds (30 for FHLS and 6 for FRCI). However, no such limitation was imposed on the lower bound. Therefore, the CIs for the model-based estimates may include values below the minimum plausible score. A total of 110 participants (57 in the control group and 53 in the intervention group) were included in baseline and follow-up FHLS total score primary analyses. Three additional participants had sufficient data for baseline and follow-up FRCI total score secondary analyses. Therefore, a sample size of 113 (59 in the control group and 54 in the intervention group) was reported for all FRCI results.

After adjusting for baseline covariates, the primary tobit regression model did not detect a significant intervention effect (0.9; 95% CI, −0.3 to 2.2; *P* = .15) (Table 3). The interaction coefficient in the post hoc, secondary interaction model indicated that the increase in the magnitude of the intervention effect for each 5-point decrease in baseline FHLS score was nonsignificant (1.3; 95% CI, −0.2 to 2.7; *P* = .09). Model-based estimates demonstrated a significant association for baseline FHLS scores from 6 to 23 (indicated by regions where the 95% CI did not include zero in Figure 2A). For context, 27% of the sample had baseline scores in the significance range.

**Table 3.** Primary and Secondary Outcomes Tobit Regression Results

Outcome	Coefficient (95% CI)	*P* value
**Primary: 12-week follow-up FHLS total score^a^**		
Baseline FHLS total score	0.2 (0.1 to 0.4)	.004
Random assignment (VTKO only vs VTKO plus VHF)	0.9 (−0.3 to 2.2)	.15
Marital status (single, divorced, separated, or widowed vs married or living as couple)	0.0 (−1.4 to 1.4)	>.99
Parental education level (high school or less vs more than high school)	−1.6 (−3.1 to −0.1)	.03
Parent race or ethnicity (vs non-Hispanic White)		
Non-Hispanic Black	−0.1 (−1.7 to 1.4)	.85
Hispanic, any race	−2.3 (−4.6 to −0.1)	.04
Other or multiracial or multiethnic^b^	1.0 (−1.3 to 3.3)	.40
**Secondary: 12-week follow-up FRCI total score^c^**		
Baseline FRCI total score	0.7 (0.5 to 0.9)	<.001
Random assignment (VTKO only vs VTKO plus VHF)	0.4 (−0.2 to 1.1)	.17
Marital status (single, divorced, separated, or widowed vs married or living as couple)	0.1 (−0.6 to 0.8)	.70
Parental education level (high school or less vs more than high school)	−0.6 (−1.3 to 0.1)	.10
Parent race or ethnicity (vs non-Hispanic White)		
Hispanic, any race	0.8 (−0.3 to 1.9)	.16
Non-Hispanic Black	0.5 (−0.3 to 1.2)	.23
Other or multiracial or multiethnic^c^	−0.1 (−1.2 to 1.0)	.87

Abbreviations: FHLS, Family Healthy Lifestyle Subscale; FRCI, Family Resilience and Connection Index; VHF, Virtual Healthier Families; VTKO, Virtual Teaching Kitchen Outreach.

^a^ Results are from a multivariable tobit regression model with an upper limit of 30 (n = 110). A total of 110 participants (57 in the control group and 53 in the intervention group) were included in baseline and follow-up FHLS total score primary analyses.

^b^ The following racial and ethnic subcategories were included in the other or multiracial or multiethnic category: Asian, Middle Eastern, Native American, Native Hawaiian, and Pacific Islander. Because only 2 participating parents self-identified as other, this subcategory was combined with the multiracial or multiethnic category.

^c^ Results are from a multivariable tobit regression model with an upper limit of 6 (n = 113). Three additional participants had sufficient data for baseline and follow-up FRCI total score secondary analyses. Therefore, a sample size of 113 (59 in the control group and 54 in the intervention group) was reported for all FRCI results.

### Secondary Outcome

Three additional families had sufficient FRCI data for secondary analyses; therefore, 113 families (59 in the control group and 54 in the intervention group) were included for all FRCI results. Baseline FRCI scores (range, 0-6) were left-skewed, with reported means (SDs) of 4.7 (1.3) and 4.3 (1.8) in the control and intervention groups, respectively (Table 2). At 12-week follow-up, mean (SD) FRCI scores were descriptively lower in the control group (4.4 [1.5]) and higher in the intervention group (4.5 [1.5]), although this difference was not significant (0.1; 95% CI, −0.5 to 0.6; *P* = .74). Internal consistency of the FRCI was relatively low (Cronbach α = 0.67 at baseline and 0.59 at 12-week follow-up).

After adjusting for baseline covariates, the primary tobit regression model did not detect a significant intervention effect (0.4; 95% CI, −0.2 to 1.1; *P* = .17) (Table 3). However, the interaction coefficient in the post hoc, secondary interaction model indicated a significant increase in the magnitude of the intervention for each 1-point decrease in baseline FRCI score (0.4; 95% CI, 0.01 to 0.8; *P* = .047). Model-based estimates demonstrated a significant association for baseline FRCI scores from 0 to 3 (indicated by regions where the 95% CI did not include zero in Figure 2C). For context, 21% of the sample had baseline scores in the significance range.

## Discussion

Although the intervention group’s mean FHLS and FRCI scores improved from baseline to 12-week follow-up, a priori analyses of the VHF program’s effect did not demonstrate statistically significant improvements compared with the control group. Post hoc, secondary analyses found that families with low baseline scores had stronger responses to the intervention, with estimates of the intervention effect size becoming significant for FHLS and FRCI baseline scores between 6 and 23 and 0 and 3, respectively. However, these analyses were not prespecified, and the results should be viewed as providing preliminary evidence for a differential intervention effect, which should be confirmed in future studies. To our knowledge, this is the first RCT to assess the effectiveness of a virtual behavioral health intervention that engages both the parent and child and targets family-level constructs of health and resilience.

Given that parents were more permissive regarding unhealthy behaviors during COVID-19 (eg, allowing increased screen time),^45,46,47^ the brief VHF sessions may not have sufficiently motivated families to break certain habits. Although families received a text message with their individualized goal immediately after a completed session, there was no additional contact with the health coach until the next session. Future iterations of this program should consider sending further reminders or including telephone calls with the health coach to help families resolve ambivalence. A previous study^48^ found that these additional opportunities for reinforcement may increase participants’ feelings of accountability and promote behavior change.

If the interactions between baseline scores and intervention effectiveness were confirmed, it would suggest that a tailored approach for family health and resilience programs might be most effective. This could be achieved by screening for level of need before enrollment. Such a personalized approach is consistent with the increasing interest in precision medicine, which seeks to develop “prevention and treatment strategies that take individual variability into account.”^49,50^ Although the application of precision medicine techniques in a behavioral health context has been limited, early research is promising.^51,52^ In a study by Rodriguez et al,^52^ patients who received a tailored intervention according to their individual readiness to eat healthier had significantly greater improvements in diet compared with patients who received a nontailored intervention. Results of the current study align with this research and provide valuable insights for future studies that aim to move beyond the traditional one-size-fits-all approach for behavioral interventions.

Although many studies indicate the need for higher contact hours for behavior change,^53,54^ the VHF program had a pragmatic approach for busy families and may have achieved effectiveness for those most in need, even with fewer contact hours delivered. For example, the US Preventive Services Task Force recommends 26 contact hours over 2 to 12 months to change behavior associated with reducing obesity,^53^ but less than 2% of patients achieve this.^55^ By allowing families to choose when to meet, including evening hours and weekends, the VHF program was designed knowing that families with young children may have difficulty engaging with intensive programs, especially during the pandemic.^6,56^ Despite only 7 families (13%) attending all 12 sessions, there is some evidence that the VHF program effectively improved health and resilience for families with lower baseline scores. Although it may be that “more is better,” these results indicate that perhaps “less is sufficient.” However, further study of the impact of varying number of sessions by design is necessary.

A notable characteristic of this RCT was the family-based approach, which engaged both the parent and child in the VHF program. This approach recognizes the bidirectional influence that parents and children have on one another’s health^57,58^ and is consistent with recommendations by the American Academy of Pediatrics and National Academy of Medicine.^59,60^ Although previous family-based interventions have demonstrated improvements in child diet and physical activity,^61,62^ these interventions have solely assessed behavior change at the individual level of the parent or child, despite the central role the family has in producing health. The present study addresses this gap by measuring family-level constructs of health and resilience. Given the novelty of this approach, further evaluation may be warranted to identify optimal measures of these constructs in family-based interventions.

### Limitations

The trial was conducted at a single site, reducing generalizability of results. The small sample size limited the ability to detect small intervention effects. Furthermore, the interaction effect analyses were not prespecified, and the study was likely underpowered to detect them. However, these concerns are partially mitigated by adherence to best practices for reporting this type of result,^63^ including transparency about how the analytic sample was obtained and encouraging replication of the post hoc result in future studies among other practices. Although it is possible that regression to the mean could have contributed to the interaction results, the RCT design of the study should mitigate these concerns. The left skew of baseline scores and ceiling effects may have limited the ability to detect improvement over time despite the use of tobit regression models. Furthermore, the active comparator condition (the VTKO program) could have contributed to improvement across both groups, which would have further limited the ability to detect effects from the VHF intervention. The trial also occurred during the expanded child tax credit, which could have contributed to higher outcome scores. However, the RCT design should have limited the potential for this tax credit to bias comparative results. Additionally, this study was limited by using self-reported measures.

## Conclusions

In this RCT of families with young children, there were no significant intervention effects in the prespecified primary analyses. However, post hoc, secondary analyses provided preliminary evidence that this innovative virtual health coaching program may have bolstered family health and resilience for families who needed it most during the COVID-19 pandemic. Understanding how to tailor behavioral health interventions according to families’ specific needs is an important next step for advancing family health and resilience.

## Notes

Supplement 1.
Trial Protocol

Supplement 2. **eTable 1.** Overview of the VHF Curriculum **eTable 2.** Family Healthy Lifestyle Subscale **eTable 3.** Family Resilience and Connection Index **eTable 4.** Comparison of Demographic Characteristics by Analysis Group

Supplement 3.
Data Sharing Statement
