# Supplementary material for: Effect of a Virtual Home-Based Behavioral Intervention on Family Health and Resilience During the COVID-19 Pandemic: A Randomized Clinical Trial
Source: JAMA Netw Open. 2022 Dec 20;5(12):e2247691. doi: 10.1001/jamanetworkopen.2022.47691 (PMC9856707; doi:10.1001/jamanetworkopen.2022.47691)
Supplement: Supplement 1. — Trial Protocol [file jamanetwopen-e2247691-s001.pdf]

VUMC Institutional Review Board

TRIAL PROTOCOL

Study Title: Food for Thought
IRB No.: 200257

Principal Investigator:
Shari L. Barkin, MD, MSHS
William K Warren Endowed Chair and Professor of Pediatrics
Division Chief of General Pediatrics
Vanderbilt University Medical Center

TABLE OF CONTENTS

1. Background……………………………………………………………………………3
2. Study Aim……………………………………………………………………………..4
3. Study Hypothesis……………………………………………………………………...4
4. Study Population and Sample Size……………………………………………………4
5. Screening and Enrollment……………………………………………………………..4
6. Study Design, Procedures, and Schedule of Events for Study Participants…………………...6
7. Incentives for Participation…………………………………………………………..11
8. Randomization……………………………………………………………………….11
9. Statistical Analysis Plan……………………………………………………………...11
10. Plans for Assuring Data Accuracy and Protocol Compliance………………………………11
11. Privacy/Confidentiality Issues……………………………………………………….12
12. Adverse Events or Unanticipated Problems Involving Risk to Participants…………………12
13. Proposal Process to Date……………………………………………………………..13
14. References…………………………………………………………………………....14

1. Background:

Food insecurity is defined as limited or uncertain access to nutritionally adequate, safe, and acceptable foods due to financial resource constraint.[1] Over fourteen million, or eleven percent, of U.S. households were food insecure at some time during 2018.[2] Rates of food insecurity in 2018 were higher than the national average for households with children, specifically children under age 6 (14.3%) and in low-income households.[2] Food insecurity in children is associated with increased risks of lower nutrient intake, cognitive problems, anemia, asthma, aggression and anxiety as well as risk of being hospitalized and reporting being in fair or poor health.[3] Additionally, it may exacerbate the onset or persistence of other adverse health conditions, including overweight and obesity.[4]

One protective factor against food insecurity is child participation in preschool programs, such as Head Start (HS), and participation in summer programs[5] HS promotes school readiness for children in low-income families by offering educational, nutritional, health, social, and other services to over 1 million children each year nationwide.[6] Also, Head Start and full-day summer programming promotes food security by providing free breakfast and lunch. Participants are from low-income families with the majority of families representing racial/ethnic minorities.[6] Food security improves both health and learning in childhood, however, there are no program regulations for routinely assessing or responding to food insecurity among HS and summer parks and recreation families.[7] In Davidson County, the Metro Action Commission provides food during the school year for HS children and for summer parks and recreation programs that reach hundreds of underserved children who can lack access to food during the summer months. Moreover, while all these meals meet USDA requirements, often food provided can include foods with high added sugar with high total calories and can be wasted.

The “Food for Thought” study will leverage the Nashville Collaborative, a well-established community-academic partnership led by Dr. Barkin, that addresses childhood obesity and the social, economic and policy-based structures associated with health disparities in obesity, including food security. Our Community Advisory Board (comprised of 15 organizations including Head Start, Parks and Recreation, the United Way, and the Nashville Public Library) recognized food security as a fundamental need for children and endorsed the project. Utilizing a Nashville Collaborative previously tested project to build cooking skills for children and parents, we will work with our community partners to design an alternative way to deliver this cooking skills program.

Due to the COVID-19 pandemic, this issue has gained even more importance. Food insecurity has increased substantially with children out of school and without in-person programming that typically would have provided breakfast and lunch. To this is the added challenge of maintaining physical distancing and ensuring the safety of underserved families who have been hit the hardest. Due to this unprecedented circumstance, the Food for Thought Steering Committee along with the Nashville Collaborative Advisory Committee, representing more than 15 different community agencies, have guided us to revise our Food for Thought grant aims. We summarize

these revised methods to continue to the same overarching goal of Food for Thought, examining how we can maximize good nutrition that is economically sustainable and influences larger related policies.

2. Study Aim:

Develop and test programs to measurably improve family health upon two rigorously tested programs adapted to the COVID-19 context. This will be done by delivering an adapted Teaching Kitchen Outreach program[8] (weekly groceries and recipe videos) as well as testing an adapted Healthier Families program[9,10] (an evidence-based 12-week health coaching intervention).

3. Study Hypothesis:

All parent-child dyads who participate in the study will have improved family health post-intervention. They will also exhibit improvements in all other outcomes measured (food security, parent and child nutrition, family resilience, etc.). Participants in the intervention group will demonstrate augmented improvements in these outcomes compared to the control group.

4. Study Population and Sample Size:

In this randomized clinical trial, rolling recruitment will occur for approximately months starting in February of 2021 and running through May of 2021 until up to 200 participating parent-child dyads that fit the eligibility inclusion criteria as outlined below have been identified. Eligible participants whose providers have given permission will be contacted by key study personnel through email, text, or phone calls to invite them to participate in a study using a standard script. We will continue to contact eligible patients until we reach our recruitment goal. Participants will be recruited from the Vanderbilt Pediatric Primary Care Clinic or Nashville Collaborative community partner sites. This study will serve underserved populations, with limited access to financial resources. Low-income households, single-parent households, and households headed by Black or Latino adults - groups historically impacted by health disparities - experience a higher prevalence of food insecurity and reduced access to healthy foods, and they are thus appropriate targets for this study. These are also the populations that primarily comprise the populations served by Head Start, Parks and Recreation, Nashville Public Library, Project Transformation, Martha O'Bryan, and St. Luke's. Of those participants that meet eligibility and are enrolled in the study, all potential benefits and burdens are distributed fairly.

5. Screening and Enrollment:

Parents will be identified by 1 of 2 processes. This will vary based on whether said potential participants are eligible to participate due to involvement with specific community partners or due to being a current patient of the Vanderbilt Primary Care Pediatrics Clinic.

1) **Community Organization Procedure**

Research staff will not be initiating contact with any participants from community partners. Community partners will share study contact information to allow interested families to contact KSP if they are interested. Partners are unable to provide a list of patron names without compromising confidentiality and their relationships with the families they serve. Partners will share our information only and will not conduct recruitment activities directly.

2) **Vanderbilt Primary Care Pediatrics Clinic Procedure**

Appropriately trained key study personnel will pre-screen patient-families by pulling electronic medical records from Epic (eStar). Parents who are contacted will also complete an eligibility screen in REDCap either verbally (with study personnel over the phone) or electronically (on their own using a computer or cell phone with internet access).

Parents and their children will be assessed for eligibility prior to informed consent and baseline data collection. Trained and certified data collectors will determine an interested parent-child dyad's eligibility according to the following criteria:

Parent eligibility inclusion criteria:

- Currently participating in programming with one of the involved community partners, including, Parks and Recreation, Nashville Public Library, Project Transformation, Head Start, Martha O'Bryan, and St. Luke's; Patients who receive primary care at the Vanderbilt Primary Care Pediatrics Clinic are eligible as well.
- Community partners will share study contact information to allow interested families to contact KSP if they are interested.
- Partners are unable to provide a list of patron names without compromising confidentiality and their relationships with their families. Partners will share our information only and will not conduct recruitment activities directly.
- Parent/legal guardian age ≥ 18 years
- Parent legal guardian of a child ages 3-8
- Ability to participate on a virtual platform (such as Zoom, FaceTime, What's App, Google Hangouts)
- Ability to attend a Zoom tool training and complete a baseline survey prior to programming
- Be able to access virtual programming through online platforms, including YouTube
- Speak English or Spanish

Parent eligibility exclusion criteria:

- inability to complete data collection measures via telephone, Redcap, or paper measures
- language other than English or Spanish

6. Study Design, Procedures, and Schedule of Events for Study Participants

Research activities will include surveys administered by trained key study personnel to measure participant demographics, maternal-child health, family coping, specifically in relation to COVID-19, and family health. Please reference Table1 for additional details including the process measures that will be recorded. All data, including data referencing a child, will be collected directly from the parent or guardian. This will include a total of 2 surveys, with one to be administered pre and post study activities. The intervention will be conducted with families of children participating in various community programs and/or receiving care from providers in the Vanderbilt Department of Pediatrics. The goal of this study is to conduct a randomized clinical trial to test the effect of two evidenced based programs (Healthier Families and Teaching Kitchen Outreach [TKO]) adapted to a virtual platform on improving family health.

Table 1: Outcomes Measured

Outcome of Interest	Source & Description
Demographics and Other Basic Information	Developed by the study team. Includes questions that capture participating parent-child dyad data regarding race/ethnicity, age, and sex.
Family Resilience and Connection	To measure the parent-child relationship in the face of stress and adversity, we will use Bethell, et al.'s six-item scale assessing family resilience, parent-child connection, and parent coping. The four family resilience index items are adapted to include additional response options to capture greater nuance. In the original scale, there is only one checkbox response option provided ("all of the time.") In our questionnaire, we will include the additional response options of "Sometimes" and "None of the time."[11]
Family Health	The Family Health Scale developed by Crandall et al. was used to measure global family health. The survey includes four subscales: 1) family social and /emotional health processes, 2) family healthy lifestyle, 3) family health resources, and 4) family external social supports and a short form to measure global family health. Our study includes the validated 32-item long-form version of the FHS.[12]
Parent and Child General Health	This component of the survey will be included to ask the participating parent's views on their own as well as their child's general health. We will solely include the first item found on the validated SF-12 Health Survey to assess this construct. We ask the question twice, though we will adapt the second iteration to reference the index child's health.
Nutrition	To assess the index parent's food and drink intake, we will include the brief eight-item Starting The Conversation (STC) Scale developed by Paxton, et al.[13] This survey has been validated in previous literature and no adaptations will be made. To assess child nutrition, the longer, in-depth Child Dietary Screener developed by the Centers for Disease Control will be used.[14]

Sleep	The Pittsburgh Sleep Quality Index will be used to measure the participating parent's quality and patterns of sleep.[15] We will include 3 items of the original 24-item long survey. The survey has been previously tested and validated by other research studies. To assess the index child's quality and patterns of sleep, Owens et al.'s Children's Sleep Habits Questionnaire will be used. This psychometrically validated, 8-item survey will be included without any adaptations.[16]
Food Patterns, Habits, and Behaviors	We will gather information on the parent-child dyad's food patterns and behaviors by utilizing relevant questions from the updated 2015-2016 version of the National Health and Nutrition Examination Survey. Specifically, we will include survey items from the Flexible Consumer Behavior Survey. To measure this same construct, we will also include items from the CDC's Youth Physical Activity and Nutrition Survey.[17]
Household Food Security	To assess financially based food insecurity and hunger, we will use a short form of the U.S. Department of Agriculture Household Food Security Scale that was developed by Blumberg, et al. This scale includes 6 of the 18 items that were a part of the original scale.[18]
COVID-19 Impact	To understand the extent to which index parents and their children are exposed to the impact of the COVID-19 pandemic, we will include the COVID-19 Exposure and Family Impact Scale (CEFIS) developed by Kazak, et al. This scale has been tested and validated by previous research studies. Part 3 of the CEFIS will be excluded from our survey.[19]

Process Measures:

Process measures will be recorded for each scheduled session of the health coaching intervention and will also include TKO recipes prepared, session duration, modules completed, goals set, progress on goals, and parent and child health coach session attendance. Health coaches will record process measures following each scheduled session, regardless of whether the session was successfully completed (i.e., parent-child dyad canceled, rescheduled, or did not show for the session).

Both the intervention and control condition will receive up to 12 weekly short (about 2-3 minutes) TKO videos. Recipe specific groceries will be delivered to participants' homes by a third-party grocery delivery company or will be available for pickup at a central location(s). Please reference Table 2 to see the recipes that will be provided as part of this intervention. These recipes reflect foods that can be purchased with SNAP and WIC and include tested meals and snacks that were developed and evaluated in person in Parks and Recreation after-school programming (Heerman W, Elsakary Y, Sommer E, Escarfuller J, Barkin S. Assessing the Scale and Spread of an Experiential Teaching Kitchen in After-School Programming Among School-Aged Children) through the long-standing Nashville Collaborative partnership (https://www.childrenshospitalvanderbilt.org/program/nashville-collaborative).[8] Over more than a decade, we have developed and tested programs together to improve health and wellness and reduce childhood obesity in our community.

Table 2. TKO Recipes and Associated Groceries

Number & Recipe	Associated Groceries to be Delivered

1. Banana Oatmeal Cookies	• 2 medium bananas<br>• 1 container of raw oats<br>• 1 container of cinnamon
2. Fruit Kabobs	• 1 whole pineapple (or 2 cans of pineapple chunks in juice)<br>• 1 orange<br>• 1 bag of grapes<br>• 1 carton of strawberries<br>• 1 pack of wooden skewers
3. Spinach Balls	• 1 bag of spinach (frozen or fresh)<br>• 1 box of whole wheat saltines<br>• 1 bag of parmesan cheese<br>• 1 small carton of eggs<br>• 1 bottle of olive oil<br>• 1 container of garlic powder
4. Fruit Pizzas	• 1 bag of medium whole wheat tortillas (minimum 6)<br>• 1 large container of plan, nonfat, Greek yogurt<br>• 1 container of strawberries<br>• 1 bag of grapes<br>• 1 can of mandarin oranges canned in juice<br>• 1 can of pineapple canned in water<br>• 1 container of vanilla extract<br>• 1 jar of honey<br>• 1 orange
5. Zucchini Pizza Boats	• 6 whole zucchinis<br>• 1 clove of garlic<br>• 1 bag of part skim mozzarella<br>• 1 bag of parmesan cheese<br>• 1 container of oregano<br>• 1 container of basil<br>• 1 container of salt<br>• 1 container of pepper<br>• 1 bottle of white grape juice<br>• 1 lemon<br>• 1 small can of tomato paste (minimum 3 oz.)
6. Blueberry Peach Oatmeal	• 2 large peaches<br>• 1 pint of blueberries
7. Cinnamon Roasted Sweet Potatoes	• 2 sweet potatoes
8. Salad Bar with Dressing	• 1 bottle of apple cider vinegar<br>• 1 bottle of low-sodium soy sauce<br>• 1 container of honey<br>• 1 container of ground ginger<br>• 1 bag of spinach

	• 1 bag or head of Romain lettuce<br>• 1 cucumber<br>• 1 box of Cherry tomatoes<br>• 1 bell pepper<br>• 1 red onion<br>• 1 bag of shredded carrots<br>• 1 bag of low-fat cheese<br>• 1 can of black beans (no salt added)<br>• 1 apple<br>• 1 box of berries (such as raspberries, blueberries, or strawberries)
9. Chicken Taco Burger	• 3 packs (1 lb. each) of lean ground chicken<br>• 1 jalapeno<br>• 1 bunch of cilantro<br>• 2 limes<br>• 1 bunch of scallions<br>• 1 jar of minced garlic<br>• 1 red onion<br>• 1 package of whole wheat buns (minimum 6)<br>• 1 head of lettuce<br>• 1 large tomato
10. Black Bean and Corn Salad	• 2 cans of corn (no salt added)<br>• 1 large cucumber or 2 small cucumbers<br>• 1 bunch of chives<br>• 1 can of black beans (no salt added)<br>• 1 bag of corn tortillas (not chips)<br>• 1 container of cumin
11. Build Your Own Banana Split	• 5 large bananas<br>• 1 large container of Greek yogurt, plain, nonfat<br>• 1 container of blueberries<br>• 1 container of blackberries<br>• 1 container of raspberries

Those randomized to the intervention condition will also receive a 12-weekly health coach via a virtual platform (such as Zoom, FaceTime, or What's App) to provide an adapted version of the previously tested Healthier Families program. Adaptations include shortening each session to 30 minutes and delivering the programming via a virtual platform. The health coach will provide the Healthier Families modules (refer to Table 3) either to individual child-parent pairs or with groups of parents (virtual group visit model). Participants will be encouraged to share pictures or videos of what they are learning and applying in their families related to the modules (e.g., recipes, cooking, physical activity). To do this, platforms such as Facebook might be used. If Facebook will be used in any form or fashion, it will be completely voluntary, and participants will be made aware that it is not a secure platform. This will be included in the consent and will be emphasized when the programming

begins as well. Any use of Zoom for participant participation will use a version of Zoom procured through Vanderbilt. This programming has been developed and evaluated in partnership with Parks and Recreation with over 600 parent-child dyads and has been shown to have a statistically significant effect on absolute BMI (Barkin SL, Heerman WJ, Sommer EC, Martin NC, Buchowski MS, Schlundt D, Po'e EK, Burgess LE, Escarfuller J, Pratt C, Truesdale KP, Stevens J. Effect of a Behavioral Intervention for Underserved Preschool-Age Children on Change in Body Mass Index: A Randomized Clinical Trial).[9]

Table 3. Adapted Healthier Families Intervention Modules and Key Messages

**Session Number & Theme**	**Key Messages**
1. Plan for Success	1. Getting to Know You<br>2. How to turn "I want" into "I will"<br>3. Connect to Community Resources
2. Choose Healthy Foods	1. Make Healthy Choices<br>2. Eat More Fiber and Less Sugar<br>3. Eat 5 Servings of Fruits and Veggies a Day
3. Be an Active Family Together	1. Be Active Together<br>2. Keep Track of What You Do<br>3. Make it Fun
4. Plan Healthy Meals	1. Plan Your Meals in Advance<br>2. Find Healthy Foods in the Store<br>3. Get the Most Bang for Your Buck<br>4. Reduce Food Waste
5. Healthy Snacks and Drinks	1. Smart Snacking<br>2. Re-Think Your Drinks<br>3. Be Sugar Smart
6. Learning How to Unplug from Media and Screens	1. Family Time Without Screens<br>2. Family Activities and Games
7. Mindful Eating	1. Knowing When to Eat<br>2. Take Your Time: How to Slow Down and Enjoy Your Food<br>3. Plan Ahead for Meal and Snack Times
8. Engaged Parenting	1. Be an Engaged Parent in a Distracted World<br>2. Be the Mirror – How to Be a Healthy Role Model<br>3. Managing Anxiety for Parents and Kids
9. Eat Together	1. Healthy Plate, Healthy Family<br>2. Eat Together as a Family<br>3. Eat a Rainbow of Fruits and Vegetables
10. Find a Fitness Home	1. Bringing Fitness to Your Home<br>2. Using Your Home Inside and Out<br>3. Using Parks and Greenspaces for Family Activity, While Staying Safe
11. Sleep Matters	1. Sleep Matters: Learn Why it is Important for You and Your Child<br>2. Know How Much Your Child Should Sleep<br>3. Plan Ahead and Keep Track

12. Maintaining and Sustaining Healthy Family Habits	1. Looking Back<br>2. Looking Forward<br>3. Continuing to Use Your Resources

7. <u>Incentives for Participation:</u>

Participating parents will receive a $15 gift card for each of the 2 surveys (baseline and 12-week follow-up), for a total of up to $30. In addition, parents will also receive all receive ingredients necessary to prepare recipes as outlined in the attached Teaching Kitchen outreach program. All ingredients will be delivered to participants' homes by a credible and current home grocery delivery program used by Vanderbilt University Medical Center, such as Shipt or Instacart. Both the adapted Teaching Kitchen Outreach and adapted Healthier Families programming will be provided at no cost to the participating parent-child dyads.

8. <u>Randomization:</u>

Randomization will occur after obtaining informed consent as well as the completion of certain baseline data collected by the parent (i.e., the baseline survey). This process will be completed within a maximum of a 30-day period. Once successfully completed, randomization will occur. Within the first cohort of the trial, parent-child dyads will be grouped according to parent preferred language (English versus Spanish), resulting in two strata within each region. Dyads within the strata will be randomized to the intervention and control groups within each of the two regions for a total of up to 200 parent-child dyads. Participants will be randomized by an unblinded KSP. Participants will be told which group they were randomized to.

9. <u>Statistical Analysis Plan:</u>

We will describe participant characteristics using mean (standard deviation [SD]) or frequency (percentage), as appropriate. Comparison of descriptive variables between the control group and intervention group will be performed using a chi-squared test for categorical variables and a 2-sample, independent t-test for continuous variables. We will test for changes between baseline and 12-week follow-up outcomes using paired t-tests for within group comparisons. We will test for changes between baseline and 12-week follow-up outcomes using 2-sample, independent t-tests for between-group comparisons of the intervention and control group. We will conduct Tobit regression models for scales in which there is a considerable degree of skewed data.

10. <u>Plans for Assuring Data Accuracy and Protocol Compliance:</u>

The principal investigator and study coordinator will be responsible for the regular evaluation of protocol compliance. This will be discussed at weekly team meetings and any deviations from the protocol will be reported to the IRB. In addition, we have developed several weekly and quarterly checks to evaluate protocol compliance with informed consent (double-checking correct signatures after collected on staff informed consent and opt-out forms and

calls received on child participants, reporting back on experience of data collectors collecting consent), data collection, and an adverse event report system

- **Survey Data**

  REDCap will be used as the primary method for data collection of survey data. The database will be developed using logic models that will ask for reasons for incomplete or missing data (e.g., not available, illegible). Online surveys will be self-administered with assistance from trained staff available to facilitate appropriate data entry and to minimize any potential literacy limitations. Paper surveys will be administered to participants by trained study team members to facilitate appropriate data entry and to minimize potential literacy limitations. Surveys will be administered to participants by key study personnel, either via phone, on paper, or via online redcap survey link depending on participant preference and feasibility.

11. <u>Privacy/Confidentiality Issues:</u>

Privacy and confidentiality are of the highest priority, and all efforts will be made to keep personal information and research records private throughout this study. Records and documents pertaining to the conduct of this study will be kept in HIPAA-compliant, secure, password-protected databases (i.e., REDCap) or in locked files accessible only by a limited number of key study personnel that have been granted access by the principal investigator. All participant data will be coded using a unique study ID. No PHI will be shared outside of the key study personnel at each institution and no PHI will be shared between institutions. The information from the research study may be published using aggregate results; however, no individual subject will be identified.

12. <u>Adverse Events or Unanticipated Problems Involving Risk to Participants:</u>

The principal investigator and research staff (with oversight by the principal investigator) are responsible for ensuring protocol compliance, data integrity, and participant safety. This protocol presents minimal risks to participants and adverse events or other problems that are not anticipated. All suggested dietary changes are evidence-based and healthy. We expect tangible benefits to accrue to all subjects of the study: participants are expected to experience improved nutrition and health as a result of participating in the study. If any physical injury or illness should occur as a direct result of participation in this study, Vanderbilt University Medical Center maintains limited research insurance coverage for the usual and customary medical fees for reasonable and necessary treatment of such injuries or illnesses. The informed consent document will include this statement and will provide pertinent contact information. In the unlikely event that such events occur, unanticipated problems involving risks to subjects or others will be reported immediately to the principal investigator and to the IRB and in writing within 7 days. While this study is not a federally funded research project (funded by the Joe C Davis Foundation), the study team will report any action resulting in the temporary or permanent suspension of this study to the donor responsible for the gift funding this study

13. Proposal Process to Date:

- Initial application submitted to the Vanderbilt University Medical Center (VUMC) Institutional Review Board (IRB) submitted on February 7, 2020
- IRB approval obtained March 12, 2020
- Final revision to IRB application on June 24, 2021
- Clinical trials registration submitted March 25, 2022
- Clinical trials registration approved on April 13, 2022. Assigned ClinicalTrials.gov Identifier: NCT05328193

14. References:

1. USDA ERS - Food Security in the U.S. Accessed February 7, 2020. https://www.ers.usda.gov/topics/food-nutrition-assistance/food-security-in-the-us/

2. Coleman-Jensen A, Rabbitt MP, Gregory CA, Singh A. Household Food Security in the United States in 2018. Published September 4, 2019. Accessed February 7, 2020. http://www.ers.usda.gov/publications/pub-details/?pubid=94848

3. Schroeder K, Smaldone A. Food insecurity: A concept analysis. *Nurs Forum (Auckl).* 2015;50(4):274-284. doi:10.1111/nuf.12118

4. Pan L, Sherry B, Njai R, Blanck HM. Food Insecurity Is Associated with Obesity among US Adults in 12 States. *J Acad Nutr Diet*. 2012;112(9):1403-1409. doi:10.1016/j.jand.2012.06.011

5. U.S. Department of Agriculture. USDA's National School Lunch Program Reduces Food Insecurity. Published August 17, 2017. Accessed February 7, 2020. https://www.ers.usda.gov/amber-waves/2017/august/usda-s-national-school-lunch-program-reduces-food-insecurity/

6. U.S. Department of Health & Human Services. Head Start. Published July 30, 2020. Accessed February 7, 2020. https://www.acf.hhs.gov/ecd/early-learning/head-start

7. American Academy of Pediatrics Council on Community Pediatrics. Promoting Food Security for All Children. *Pediatrics*. 2015;136(5):e1431-e1438. doi:https://doi.org/10.1542/peds.2015-3301

8. Heerman WJ, Elsakary Y, Sommer EC, Escarfuller J, Barkin SL. Assessing the scale and spread of an experiential teaching kitchen in after-school programming among school-age children. *Public Health Nutr*. 2021;24(12):3937-3944. doi:10.1017/S1368980020004206

9. Barkin SL, Heerman WJ, Sommer EC, et al. Effect of a Behavioral Intervention for Underserved Preschool-Age Children on Change in Body Mass Index: A Randomized Clinical Trial. *JAMA*. 2018;320(5):450-460. doi:10.1001/jama.2018.9128

10. Po'e EK, Heerman WJ, Mistry RS, Barkin SL. Growing Right Onto Wellness (GROW): A Family-Centered, Community-Based Obesity Prevention Randomized Controlled Trial for Preschool Child-Parent Pairs. *Contemp Clin Trials*. 2013;36(2):10.1016/j.cct.2013.08.013. doi:10.1016/j.cct.2013.08.013

11. Bethell CD, Gombojav N, Whitaker RC. Family Resilience And Connection Promote Flourishing Among US Children, Even Amid Adversity. *Health Aff (Millwood).* 2019;38(5):729-737. doi:10.1377/hlthaff.2018.05425

12. Crandall A, Weiss-Laxer NS, Broadbent E, et al. The Family Health Scale: Reliability and Validity of a Short- and Long-Form. *Front Public Health*. 2020;8:587125. doi:10.3389/fpubh.2020.587125

13. Mâsse LC, O'Connor TM, Lin Y, et al. The physical activity parenting practices (PAPP) item Bank: a psychometrically validated tool for improving the measurement of physical activity parenting practices of parents of 5-12-year-old children. *Int J Behav Nutr Phys Act*. 2020;17(1):134. doi:10.1186/s12966-020-01036-0

14. Centers for Disease Control and Prevention (CDC). *Physical Activity Guidelines for Americans, 2nd Edition*. U.S. Department of Health and Human Services; :118. https://health.gov/sites/default/files/2019-09/Physical_Activity_Guidelines_2nd_edition.pdf

15. Shahid A, Wilkinson K, Marcu S, Shapiro CM. Pittsburgh Sleep Quality Index (PSQI). In: Shahid A, Wilkinson K, Marcu S, Shapiro CM, eds. *STOP, THAT and One Hundred Other Sleep Scales*. Springer New York; 2011:279-283. doi:10.1007/978-1-4419-9893-4_67

16. Owens JA, Spirito A, McGuinn M. The Children's Sleep Habits Questionnaire (CSHQ): psychometric properties of a survey instrument for school-aged children. *Sleep*. 2000;23(8):1043-1051.

17. Centers for Disease Control and Prevention (CDC). NHANES Questionnaires, Datasets, and Related Documentation. Accessed February 7, 2020. https://wwwn.cdc.gov/nchs/nhanes/continuousnhanes/default.aspx?BeginYear=2019

18. Blumberg SJ, Bialostosky K, Hamilton WL, Briefel RR. The effectiveness of a short form of the Household Food Security Scale. *Am J Public Health*. 1999;89(8):1231-1234. doi:10.2105/ajph.89.8.1231

19. Kazak AE, Alderfer M, Enlow PT, et al. COVID-19 Exposure and Family Impact Scales: Factor Structure and Initial Psychometrics. *J Pediatr Psychol*. 2021;46(5):504-513. doi:10.1093/jpepsy/jsab026
