# Supplementary material for: Effect of a Virtual Home-Based Behavioral Intervention on Family Health and Resilience During the COVID-19 Pandemic: A Randomized Clinical Trial
Source: JAMA Netw Open. 2022 Dec 20;5(12):e2247691. doi: 10.1001/jamanetworkopen.2022.47691 (PMC9856707; doi:10.1001/jamanetworkopen.2022.47691)
Supplement: Supplement 2. — eTable 1. Overview of the VHF Curriculum eTable 2. Family Healthy Lifestyle Subscale eTable 3. Family Resilience and Connection Index eTable 4. Comparison of Demographic Characteristics by Analysis Group [file jamanetwopen-e2247691-s002.pdf]

Supplementary Online Content

Popescu F, Sommer EC, Mahoney MR, Adams LE, Barkin SL. Effect of a virtual home-based behavioral intervention on family health and resilience during the COVID-19 pandemic: a randomized clinical trial. *JAMA Netw Open*. 2022;5(12):e2247691. doi:10.1001/jamanetworkopen.2022.47691

eTable 1. Overview of the VHF Curriculum
eTable 2. Family Healthy Lifestyle Subscale
eTable 3. Family Resilience and Connection Index
eTable 4. Comparison of Demographic Characteristics by Analysis Group

This supplementary material has been provided by the authors to give readers additional information about their work.

© 2022 Popescu F et al. *JAMA Network Open.*

eTable 1. Overview of the VHF Curriculum

Session No. and Title	Key Messages
1. Plan for Success	1. Getting to Know You<br>2. How to turn “I want” into “I will”<br>3. Connect to Community Resources
2. Choose Healthy Foods	1. Make Healthy Choices<br>2. Eat More Fiber and Less Sugar<br>3. Eat 5+ Servings of Fruits and Veggies a Day
3. Be an Active Family Together	1. Be Active Together<br>2. Keep Track of What You Do<br>3. Make it Fun
4. Plan Healthy Meals	1. Plan Your Meals in Advance<br>2. Find Healthy Foods in the Store<br>3. Get the Most Bang for Your Buck<br>4. Reduce Food Waste
5. Healthy Snacks and Drinks	1. Smart Snacking<br>2. Re-Think Your Drinks<br>3. Be Sugar Smart
6. Learning How to Unplug from Media and Screens	1. Family Time Without Screens<br>2. Family Activities and Games
7. Mindful Eating	1. Knowing When to Eat<br>2. Take Your Time: How to Slow Down and Enjoy Your Food<br>3. Plan Ahead for Meal and Snack Times
8. Engaged Parenting	1. Be an Engaged Parent in a Distracted World<br>2. Be the Mirror – How to Be a Healthy Role Model<br>3. Managing Anxiety for Parents and Kids
9. Eat Together	1. Healthy Plate, Healthy Family<br>2. Eat Together as a Family<br>3. Eat a Rainbow of Fruits and Vegetables
10. Find a Fitness Home	1. Bringing Fitness to Your Home<br>2. Using Your Home Inside and Out<br>3. Using Parks and Greenspaces for Family Activity, While Staying Safe
11. Sleep Matters	1. Sleep Matters: Learn Why it is Important for You and Your Child<br>2. Know How Much Your Child Should Sleep<br>3. Plan Ahead and Keep Track
12. Maintaining and Sustaining Healthy Family Habits	1. Looking Back<br>2. Looking Forward<br>3. Continuing to Use Your Resources

Abbreviations: VHF, Virtual Healthier Families.

© 2022 Popescu F et al. *JAMA Network Open.*

eTable 2. Family Healthy Lifestyle Subscale[a]

Survey Item	Response Options	Score
We make a point of being physically active during daily life.	Strongly Disagree<br>Somewhat Disagree<br>Neither Agree nor Disagree<br>Somewhat Agree<br>Strongly Agree	1<br>2<br>3<br>4<br>5
We usually have fresh fruits and vegetables in our home.	Strongly Disagree<br>Somewhat Disagree<br>Neither Agree nor Disagree<br>Somewhat Agree<br>Strongly Agree	1<br>2<br>3<br>4<br>5
We help each other avoid unhealthy habits.	Strongly Disagree<br>Somewhat Disagree<br>Neither Agree nor Disagree<br>Somewhat Agree<br>Strongly Agree	1<br>2<br>3<br>4<br>5
We make a point to follow medical recommendations.	Strongly Disagree<br>Somewhat Disagree<br>Neither Agree nor Disagree<br>Somewhat Agree<br>Strongly Agree	1<br>2<br>3<br>4<br>5
We help each other in seeking health care services when needed (such as making doctor's appointments).	Strongly Disagree<br>Somewhat Disagree<br>Neither Agree nor Disagree<br>Somewhat Agree<br>Strongly Agree	1<br>2<br>3<br>4<br>5
We help each other make healthy changes.	Strongly Disagree<br>Somewhat Disagree<br>Neither Agree nor Disagree<br>Somewhat Agree<br>Strongly Agree	1<br>2<br>3<br>4<br>5

[a] The Family Healthy Lifestyle Subscale was developed by Crandall et al., 2020 (https://doi.org/10.3389/fpubh.2020.587125). Scoring instructions: Six-item score (6-30) with one point for each “Strongly Disagree” response and five points for each “Strongly Agree” response. Higher scores indicate better family health.

© 2022 Popescu F et al. *JAMA Network Open.*

eTable 3. Family Resilience and Connection Index[a]

Survey Item	Response Options	Score
Family Resilience Index<br>When your family faces problems, how often are you likely to:		
talk together about what to do	All of the time<br>Sometimes<br>None of the time	1<br>0<br>0
work together to solve our problems	All of the time<br>Sometimes<br>None of the time	1<br>0<br>0
know we have strengths to draw on	All of the time<br>Sometimes<br>None of the time	1<br>0<br>0
stay hopeful even in difficult times	All of the time<br>Sometimes<br>None of the time	1<br>0<br>0
Parent-Child Connection		
How well can you and your child share ideas or talk about things that really matter?	Not very well or not at all<br>Somewhat well<br>Very well	0<br>0<br>1
Parent Coping		
How well do you think you are handling the daily demands of raising children?	Not very well or not at all<br>Somewhat well<br>Very well	0<br>0<br>1

[a] The Family Resilience and Connection Index was developed by Bethell, Gombojav, and Whitaker, 2019 (https://doi.org/10.1377/hlthaff.2018.05425). Scoring instructions: Six-item score (0–6) with one point for each "All of the time" response to the four Family Resilience Index items, and one point for each "Very well" response to the Parent-Child Connection and Parent Coping items. Higher scores indicate stronger family resilience and connection.

© 2022 Popescu F et al. *JAMA Network Open.*

eTable 4. Comparison of Demographic Characteristics by Analysis Group

Characteristic	**Participants, No. (%)** Included in Final Analyses of the Primary Outcome (n = 110)	Not Included in Final Analyses of the Primary Outcome (n = 13)	***P* value**[a]
Child			
Child age, mean (SD), y	5.2 (1.7)	5.3 (1.8)	.83
Child sex			
Male	62 (56)	7 (54)	.86
Parent			
Parent age, mean (SD), y	35.1 (8.2)	32.1 (7.4)	.26
Parent sex			
Male	6 (5)	2 (15)	.11
Parent race/ethnicity			
Black, non-Hispanic	55 (50)	8 (62)	.57
Parent education			
High school or less	29 (26)	6 (46)	.11
Parent marital status			
Married or living as couple	50 (45)	6 (46)	.96
Family			
Number of children in household			
More than one child	79 (72)	10 (77)	.70
Number of adults in household			
More than one adult	69 (63)	6 (46)	.22
Household food security			
Food insecure with or without hunger	29 (26)	5 (38)	.32
Use of SNAP and/or WIC			
Yes	62 (56)	6 (46)	.46
Household income			
Less than $10,000	21 (19)	3 (23)	.12

Abbreviations: SNAP, Supplemental Nutrition Assistance Program; WIC, the Special Supplemental Nutrition Program for Women, Infants, and Children.

[a] Calculated using a two-sample independent t-test for continuous variables and a chi-square test for independence for categorical variables.

© 2022 Popescu F et al. *JAMA Network Open.*
