# Supplementary material for: Effect of a Virtual Home-Based Behavioral Intervention on Family Health and Resilience During the COVID-19 Pandemic: A Randomized Clinical Trial
Source: JAMA Netw Open. 2022 Dec 20;5(12):e2247691. doi: 10.1001/jamanetworkopen.2022.47691 (PMC9856707; doi:10.1001/jamanetworkopen.2022.47691)
Supplement: Supplement 3. — Data Sharing Statement [file jamanetwopen-e2247691-s003.pdf]

Data Sharing Statement

Popescu. Effect of a Virtual Home-Based Behavioral Intervention on Family Health and Resilience During the COVID-19 Pandemic. *JAMA Netw Open*. Published December 20, 2022. doi:10.1001/jamanetworkopen.2022.47691

Data
Data available: No

Additional Information
Explanation for why data not available: The current study is not a federally funded project, and all datasets generated and/or analyzed are not publicly available. However, deidentified participant data may be available from the authors upon reasonable request.
